# Proteomic Analysis of Exosomes from Adipose-Derived Mesenchymal Stem Cells: A Novel Therapeutic Strategy for Tissue Injury

**DOI:** 10.1155/2020/6094562

**Published:** 2020-03-03

**Authors:** Xin Xing, Shuang Han, Gu Cheng, Yifeng Ni, Zhi Li, Zubing Li

**Affiliations:** The State Key Laboratory Breeding Base of Basic Science of Stomatology (Hubei-MOST) and Key Laboratory of Oral Biomedicine Ministry of Education, School and Hospital of Stomatology, Wuhan University, Wuhan 430079, China

## Abstract

Exosomes are extracellular membranous nanovesicles that mediate local and systemic cell-to-cell communication by transporting functional molecules, such as proteins, into target cells, thereby affecting the behavior of receptor cells. Exosomes originating from adipose-derived mesenchymal stem cells (ADSCs) are considered a multipotent and abundant therapeutic tool for tissue injury. To investigate ADSC-secreted exosomes and their potential function in tissue repair, we isolated exosomes from the supernatants of ADSCs via ultracentrifugation, characterized them via transmission electron microscopy, nanoparticle tracking analysis, and Western blot analysis. Then, we determined their protein profile via proteomic analysis. Results showed that extracellular vesicles, which have an average diameter of 116 nm, exhibit a cup-shaped morphology and express exosomal markers. A total of 1,185 protein groups were identified in the exosomes. Gene Ontology analysis indicated that exosomal proteins are mostly derived from cells mainly involved in protein binding. Protein annotation via the Cluster of Orthologous Groups system indicated that most proteins were involved in general function prediction, posttranslational modification, protein turnover, and chaperoning. Further, pathway analysis revealed that most of the proteins obtained participated in metabolic pathways, focal adhesion, regulation of the actin cytoskeleton, and microbial metabolism. Some tissue repair-related signaling pathways were also discovered. The identified molecules might serve as potential therapeutic targets for future studies.

## 1. Introduction

Mesenchymal stem cells (MSCs) are a type of multipotent stem cells with high self-renewal and multidirectional differentiation potential [1]. They are the most commonly employed cells in regenerative medicine [2, 3]. Among the different sources of MSCs, adipose tissue is perceived as an abundant and easily obtainable pool with the benefits of extensive distribution and minimally invasive extraction [4]. Adipose-derived mesenchymal stem cells (ADSCs), which are adipose-derived stromal cells originating from stromal–vascular fragments of adipose tissue, show promising therapeutic value because of their essential role in enhancing the injury repair of many tissues [5–9]. ADSCs are believed to promote tissue regeneration either directly, by differentiating into a variety of cell types, such as osteoblasts, chondrocytes, liver cells, and neuronal cells, or indirectly, via paracrine signaling, whereby secreted factors activate regenerative pathways in host tissues [10, 11].

Research on exosomes is gaining wide attention on account of the potential of these nanoscale vesicles of endocytic origin to play an important role in cell-to-cell communication and their prospective use in novel therapeutic strategies [12–14]. Exosomes represent a discrete population of vesicles secreted by multiple cells with diameters of approximately 30–150 nm [15]. These membrane-enclosed vesicles carry various functional proteins, microRNAs, and mRNAs; transfer information between cells; and mediate many physiological and pathological processes [16]. Emerging evidence has confirmed that ADSC-derived exosomes support damaged tissue repair and represent a novel therapeutic strategy to regenerate many tissue types, such as the heart, bone, cartilage, liver, nerve, and skin [17–21]. However, the specific molecular regulatory mechanisms of these effects have yet to be fully understood. Some of the regenerative properties of ADSC-derived exosomes may be associated with their internal components. Thus, understanding exosome contents is beneficial to the exploration of potential target cells and the identification of the regulatory mechanism of the ADSC paracrine signaling network. This knowledge can help in efforts to develop efficient therapeutics promoting tissue regeneration.

In this study, we isolated exosomes from mouse ADSCs and explored their morphological features and biomarkers. We then identified 1,185 proteins in ADSC-derived exosomes via liquid chromatography-mass spectrometry (LC-MS/MS). Bioinformatic analyses were performed to investigate the systematic proteomic characteristics and related signaling pathways. Our findings might expand our knowledge of the roles played by exosomes in tissue regeneration and shed new light on potential targets for treatment.

## 2. Materials and Methods

### 2.1. Isolation and Culture of Mouse ADSCs

All animal procedures were conducted with the approval of the Animal Ethics Committee of Wuhan University. Mouse ADSCs were isolated, cultured, and characterized as described in a previous study [22] with slight modifications. In brief, adipose tissues were separated from the subcutaneous inguinal region and digested with 0.2% type I collagenase (Beijing Solarbio Science & Technology Co. Ltd., C8140) at 37°C for 1 h. After filtering through 100 *μ*m cell strainers (BD Falcon) and centrifugation at 1,000 × *g* for 10 min, the remaining cells were resuspended in Dulbecco's modified Eagle medium (DMEM)/F12 (Gibco, Grand Island, NY) containing 10% fetal bovine serum (HyClone, Logan, UT) and 1% penicillin–streptomycin solution (HyClone, Logan, UT). The obtained ADSCs were incubated at 37°C in a humidified atmosphere containing 5% CO_2_. ADSCs of the third passage were used in the following experiments.

### 2.2. Multilineage Differentiation and Phenotypic Identification of ADSCs

ADSC surface biomarkers were identified via flow cytometry analysis. In brief, ADSCs were trypsinized and washed to obtain single-cell suspensions. Subsequently, the cells were incubated with the following primary antibodies at room temperature for 30 min: allophycocyanin- (APC-) labeled anti-mouse CD34 (Cat. No.: ab18225, Abcam, Cambridge, MA), phycoerythrin- (PE-) labeled anti-mouse CD44 (Cat. No.: 560569, BD Biosciences), and PerCP-labeled anti-mouse CD45 (Cat. No.: 557235, BD Biosciences). Cells incubated with isotype-matched normal IgG were used as controls, and the samples were determined via flow cytometry (FACSort, BD Biosciences).

Cells were plated into six-well plates and cultured in their respective differentiation medium to detect the differentiation ability of ADSCs. Cultured ADSCs were incubated with an osteogenic induction medium containing 50 *μ*g/mL ascorbic acid, 10 mM *β*-glycerophosphate, and 10 nM dexamethasone (Sigma-Aldrich Co., USA) for 21 days for osteogenic differentiation. Calcium deposition was analyzed via Alizarin Red staining. Cells were stained with Oil Red O 14 days post culture in adipogenic differentiation media containing 10 *μ*L/mL insulin, 1 *μ*M dexamethasone, 0.5 mM indomethacin, and 60 *μ*M 3-isobutyl-1-methylxanine (Sigma-Aldrich Co., USA). ADSCs were cultured with chondrogenic differentiation induction medium (Cyagen Co., Ltd., China) for 14 days. The fixed cells were stained with 1% (*m*/*v*) Alcian Blue 8GX (Sigma-Aldrich Co., USA) in accordance with the manufacturer's instructions.

### 2.3. Isolation of Exosomes by Ultracentrifugation

Cells were grown in the presence of serum until they reached a subconfluent state (80%–90%) at 37°C and 5% CO_2_. Then, they were gently rinsed three times with phosphate-buffered saline (PBS) and cultured with serum-free DMEM/F12 for 48 h. The cell-conditioned medium was collected and subjected to gradient centrifugation (300 × *g* for 10 min, 2,000 × *g* for 10 min, and 10,000 × *g* for 30 min) to remove residual cells and debris. Exosomes were pelleted from the supernatants through ultracentrifugation at 100,000 × *g* for 70 min by using an SW32 Ti swinging bucket rotor (Beckman Coulter, Fullerton, CA, USA). The pellets were resuspended in PBS, pooled, and ultracentrifuged once more at the same high speed for 70 min to eliminate contaminating proteins. The final exosome pellet was resuspended in PBS for further experiments.

### 2.4. Transmission Electron Microscopy (TEM)

TEM was conducted for morphological observation of the isolated exosomes. The PBS-suspended exosomes were placed on a carbon-coated copper grid (200 mesh) and allowed to adsorb onto it for 60 s. The grid was washed twice in double-distilled water and incubated in a 2% aqueous solution of uranyl acetate for 10 s. Samples were examined with an HT-7700 TEM system (Hitachi, Japan).

### 2.5. Western Blot Analysis

Western blot analysis was used to determine the characteristic surface marker proteins of exosomes. In brief, the samples were lysed in a protein extraction reagent containing a protease inhibitor. The samples were loaded onto 10% sodium dodecyl sulfate polyacrylamide gels. After electrophoresis, the proteins were transferred to a polyvinylidene difluoride membrane (Millipore) for 1 h. The membrane was blocked with 1% bovine serum albumin (Gibco) followed by incubation overnight with the following primary antibodies: anti-CD9 (Abcam, ab92726), anti-CD63 (Abcam, ab217345), and anti-TSG101 (Abcam, ab30871). After washing with TBST, the membrane was incubated with goat anti-rabbit IgG H&L (Abcam, ab205718) as a secondary antibody for 1 h at room temperature. The reaction was revealed via an enhanced chemiluminescence detection system (GE Healthcare, Little Chalfont, UK).

### 2.6. Exosome Size Analysis

The size distribution and concentration of ADSC exosomes were measured via nanoparticle tracking analysis (NTA) using a NanoSight NS300 instrument according to the manufacturer′s instructions (Malvern, UK) as previously reported [23]. The results were analyzed using the NTA analytical software (version 3.4).

### 2.7. Sample Preparation and Tryptic Digestion

Proteins were extracted from the samples with lysis buffer and added with 10 mM dithiothreitol. After sonication and centrifugation, the sample was alkylated with 55 mM iodoacetamide in the dark at room temperature. The proteins in the supernatant were analyzed via the Bradford method after centrifugation. Then, proteins were subjected to trypsin digestion at 37°C overnight. The peptides were desalted on a C18 column, dried in a vacuum concentration meter, and dissolved in 15 *μ*L of loading buffer (0.1% formic acid and 3% acetonitrile) with vortexing prior to storage at −20°C for LC-MS/MS.

### 2.8. Mass Spectrometry

Proteins were analyzed in accordance with the manufacturer's instructions. LC-MS/MS analysis was performed using an ekspert™ nanoLC system coupled online to a TripleTOF 5600-plus mass spectrometer (SCIEX, Framingham, MA, USA). Peptide was loaded onto a chromatographic capillary C18 trap column (5 *μ*m, 100 *μ*m × 20 mm) and eluted at a rate of 300 nL/min onto a C18 analytical column (3 *μ*m, 75 *μ*m × 150 mm) with a 120 min gradient by using a binary mobile phase system (buffer A: 2% acetonitrile/0.1% formic acid; buffer B: 98% acetonitrile/0.1% formic acid). Tandem mass spectrometry was conducted in a data-dependent mode with a scan resolution of 70,000 during acquisition (200 m/z). The 40 most intense precursors from a survey scan were selected for fragmentation, and MS^2^ spectra were collected over the range of 50–2,000 m/z with a scan time of 100 ms. Precursor ions were excluded from reselection for 15 s.

### 2.9. Bioinformatic Analysis

The original MS/MS file data were submitted to ProteinPilot Software v4.5 for data analysis. The sequences of the identified proteins were mapped to Gene Ontology (GO) terms to determine their biological and functional properties. A total of the three main types of annotations, namely, cellular components, molecular functions, and biological process, were obtained from the GO Consortium website. Protein annotation via the Clusters of Orthologous Groups (COGs) system was conducted in this study to annotate genes from new genomes and determine their genome evolution. Protein-enriched pathways were generated via Kyoto Encyclopedia of Genes and Genomes (KEGG) analysis and Cytoscape software 3.5.1.

### 2.10. Statistical Analysis

All experiments were performed thrice. All values were represented as the means ± standard deviations. GraphPad Prism 6.0 software was used for statistical analysis together with appropriate tests for comparisons (e.g., one-way ANOVA and Tukey's test). *P* < 0.05 was considered statistically significant.

## 3. Results

### 3.1. Characterization of ADSCs

**Figure 1:**
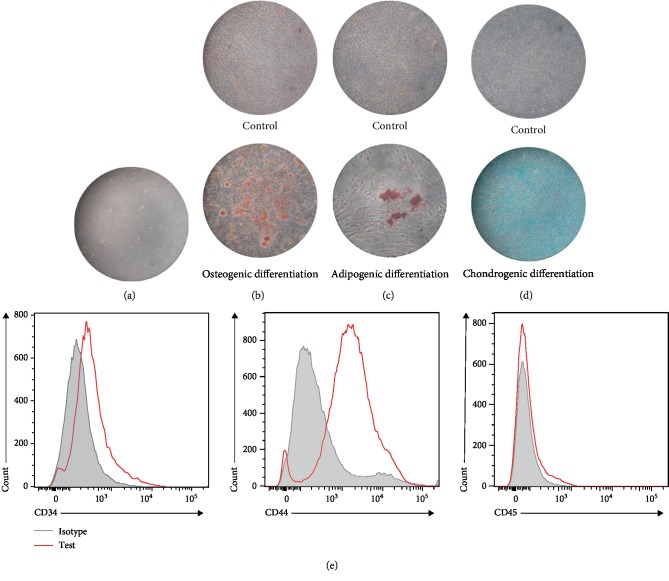
Characteristics of ADSCs. (a) The morphology of primary ADSCs (third passage) monitored by phase-contrast microscopy. (b) Osteogenic differentiation potential of ADSCs stained with Alizarin Red S. (c) ADSCs differentiated into adipocytes containing large amounts of Oil Red O-positive lipid droplets. (d) Chondrogenic differentiation of ADSCs stained with Alcian Blue. (e) Detection of the specific markers of ADSCs by flow cytometry.

ADSCs were successfully isolated from the subcutaneous fat of mice, and fibroblast-like cells were observed in the third passage of the primary culture (Figure 1(a)). Alizarin Red S, Oil Red, and Alcian Blue staining assays confirmed the multilineage potential of the isolated cells into osteogenic, adipogenic, and chondrogenic lineages (Figures 1(b)–1(d)). The flow cytometry analysis results shown in Figure 1(e) reveal that ADSCs are positive for CD44 (MSC markers) and negative for CD34 and CD45 (hematopoietic marker). These results demonstrated that the isolated cells used in the present study had typical ADSC characteristics.

### 3.2. Identification of ADSC-Derived Exosomes

**Figure 2:**
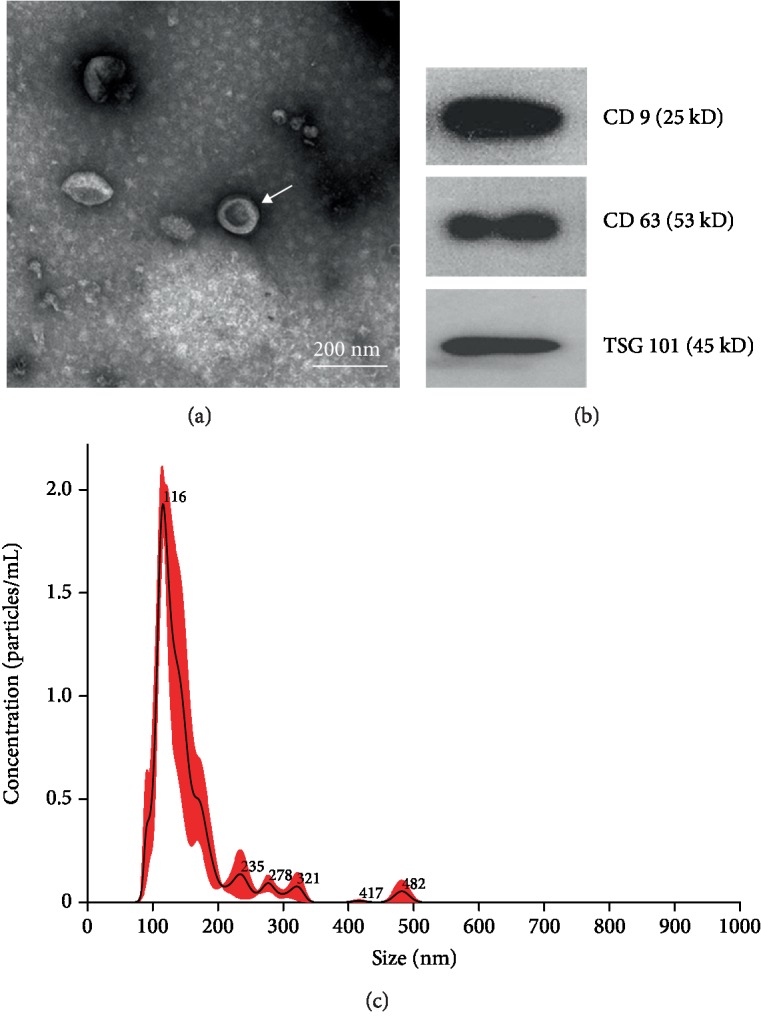
Identification of ADSC-derived exosomes. (a) TEM images of exosomes from ADSCs. (b) Expression of CD9, CD63, and TSG101 in the ADSC-derived exosomes as detected via western blot analysis. (c) Size distribution of ADSC-derived exosomes detected via nanoparticle tracking analysis.

The presence of small membrane vesicles with a typical cup-shaped structure ranging in size from 40 nm to 100 nm was captured by TEM (Figure 2(a)). The biochemical characterization of the exosomes showed the strong expression of exosomal marker proteins, including CD9, CD63, and TSG101 (Figure 2(b)). The size distribution of particles in the pellets after ultracentrifugation was measured via the NTA system. The major peak in particle size was found at 116 nm, and the overall size distribution ranged between 60 and 200 nm (Figure 2(c)).

### 3.3. Proteomic Analysis of Exosomes Derived from ADSCs

**Figure 3:**
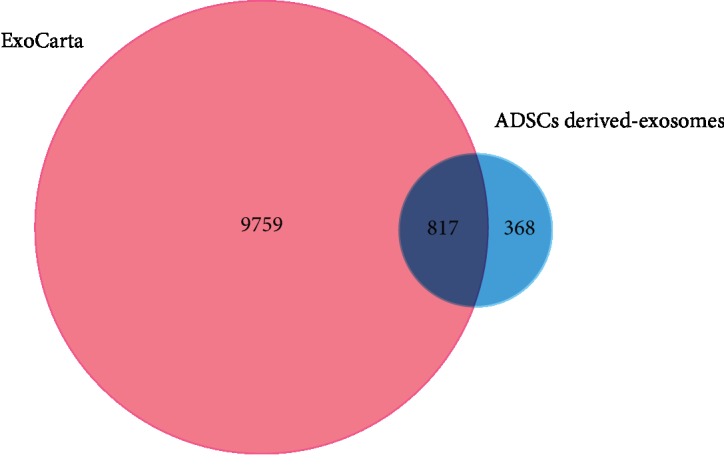
Venn diagrams of ADSC-derived exosomes against the ExoCarta database.

The peptide samples were analyzed by LC/MS/MS to profile proteins in the ADSC-derived exosomes. As shown in Figure 3(a), the software identified 1,185 proteins in all sample pairs. Among the proteins detected in the ADSC-exosome preparations, 817 (68.95%) overlapped with the results obtained from the exosome database ExoCarta. This result indicates that the procedures for isolation and purification are reproducible and that the results of proteomics analysis are reliable.

### 3.4. Functional Categories

**Figure 4:**
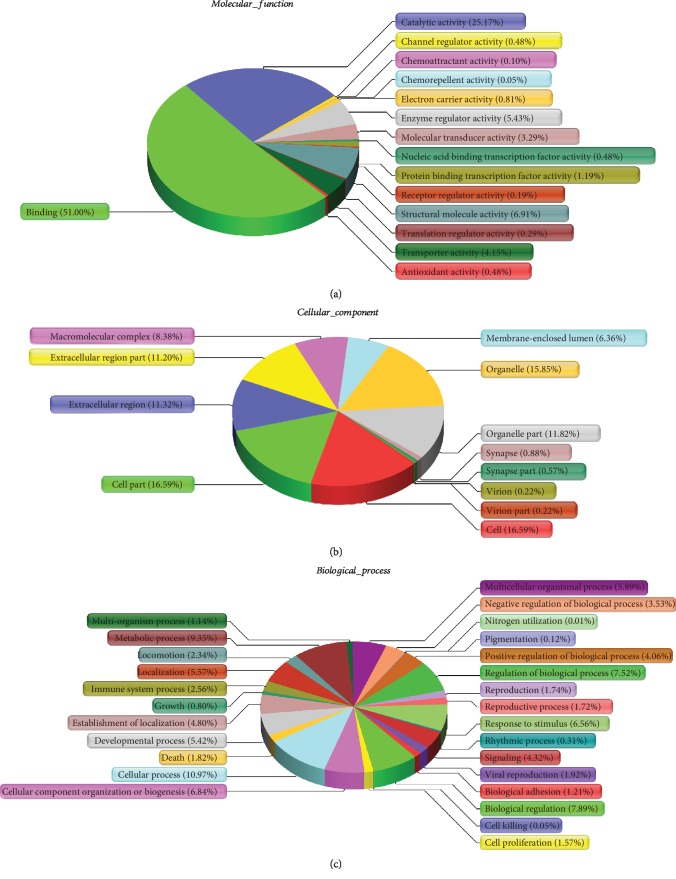
GO analysis of molecular functions, cellular components, and biological processes.

GO annotation was performed to determine the functional roles of the ADSC-derived exosome proteins and obtain enriched terms for molecular functions, biological processes, and cellular components. The GO classification system revealed that the proteins could be classified into groups based on their functional properties. For molecular functions, exosomal proteins were mainly enriched in “protein binding” and “catalytic activity” (Figure 4(a)). The cellular components most populated with exosomal proteins are “cell,” “cell part,” and “organelle” (Figure 4(b)). Biological process analysis revealed the enrichment of exosomal proteins related to “cellular process,” “metabolic process,” and “biological regulation” (Figure 4(c)).

### 3.5. COG

**Figure 5:**
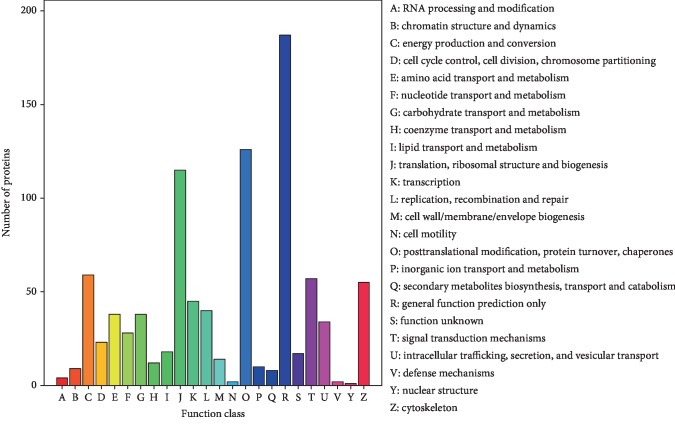
COG function classification of ADSC-derived exosomes.

The COG database was used for the functional annotation of proteins and evolutionary studies. The 708 proteins identified were assigned to 24 different COG categories. Most proteins were involved in “general function prediction only”. Other common COG functions included “posttranslational modification, protein turnover, and chaperones” (126 proteins), “translation and ribosomal structure and biogenesis” (115 proteins), “energy production and conversion” (59 proteins), and “signal transduction mechanisms” (57 proteins) (Figure 5).

### 3.6. KEGG Pathway Analysis

**Figure 6:**
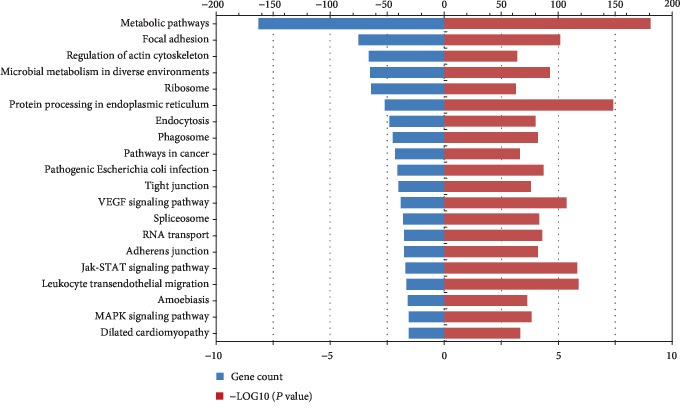
KEGG pathway analysis.

KEGG pathway analysis was performed to determine the signaling pathways enriched with the ADSC exosome proteins. A total of 822 proteins were identified via KEGG annotation and mapped to 200 pathways. As shown in Figure 6, the top 20 pathways enriched with a *P* value < 0.05 were metabolic pathways, focal adhesion, regulation of the actin cytoskeleton, microbial metabolism in diverse environments, and so on. Among these pathways, several signaling pathways, including the MAPK, VEGF, and Jak-STAT signaling pathways, are relevant to tissue injury repair [20, 24, 25].

## 4. Discussion

Exosomes are small extracellular vesicles containing functional molecules from their host cells and participating in intercellular communication [26, 27]. Among all the containing bioactive substances, nucleic acids such as microRNAs have been widely explored [28, 29]. However, as the most direct and efficient embodiment of life functions, exosomal proteins play an equally important role in signal transduction and regulation. Chen et al. reported that exosomal PD-L1 can be released from metastatic melanomas and suppress the function of CD8 T cells, thereby facilitating tumor growth [30]. Lazar et al. found that adipocyte exosomes carry proteins implicated in fatty acid oxidation which are then taken up by tumor cells, leading to increased migration and invasion [31]. Chen et al. demonstrated that exosomal DMBT1 from human urine-derived stem cells facilitates diabetic wound healing by promoting angiogenesis [32]. Abundant data have demonstrated that ADSC exosomes contribute to myocardial, hepatic, renal, skin, and bone repair and neuroprotection [33–37]. Analyzing the protein contents of exosomes in ADSCs may help improve the understanding of their functional roles in tissue repair.

Several studies have reported the proteomic signature of extracellular vesicles derived from mesenchymal stem cells (MSCs). Kim et al. identified 730 proteins in the proteomic analysis of microvesicles from human bone marrow MSCs. The microvesicle proteins contained markers of MSCs as well as signaling molecules regulating the self-renewal and differentiation capacities of MSCs [38]. La Greca et al. identified 560 proteins from pluripotent stem cell-derived extracellular vesicles through proteomic analysis. Extracellular vesicles shared 37.32% of the proteins with parental cells and enriched with molecules relevant with immunity, extracellular matrix, and cell adhesion [39]. Otero-Ortega et al. studied the proteomic signature of rat adipose-derived MSC-EVs in an experimental animal model of subcortical stroke. Proteomic analysis identified 2,416 proteins and most of them are associated with brain repair function [40]. In our work, mouse ADSC exosomes were successfully isolated and characterized. Proteomic analysis was performed, and 1,185 proteins were identified through LC-MS/MS. Bioinformatic analysis was performed to explore their potential mechanisms.

The Venn diagram produced a clear comparison of the proteomic dataset generated in this study versus other proteomic datasets described in the literature. The gene symbols of the identified exosomal proteins were compared with those in ExoCarta, a gene symbol database of published proteins identified from exosomes. Our results also showed that 817 proteins (68.95%) among our identified protein gene symbols were present in the database. Interestingly, 368 of the proteins discovered in our study were unreported.

GO analysis revealed a proteome dataset for exosomes derived from ADSCs. The cellular functions determined by GO analysis showed that the identified proteins were enriched in “cell” and “cell part,” thus indicating that the contents of exosomes share close ties with their host cells [41]. Protein binding was the most common characteristic (51%) among the molecular functions determined by GO analysis. This finding reveals that direct regulation of protein–protein interactions might be the main type of regulation conducted by ADSCs. Among the biological processes determined by GO analysis, most of the 1,185 obtained proteins were functionally related to “cellular process,” “metabolic process,” “biological regulation,” “regulation of biological process,” “response to stimulus,” and “multicellular organismal process.” Cellular processes, such as cell proliferation, differentiation, and attachment, extensively participate in tissue repair [42], similar to metabolic processes [43]. Researchers compared the proteomic of human bone marrow MSCs and their exosomes under ischemic tissue-simulated conditions and found that several putative paracrine effectors of angiogenesis include platelet-derived growth factor, epidermal growth factor, fibroblast growth factor, and most notably nuclear factor kappa B (NF*κ*B) signaling pathway proteins enriched in exosomes [44]. In a proteomic analysis of extracellular vesicles derived from pig adipose MSCs, Eirin et al. found 277 proteins enriched in extracellular vesicles versus MSCs. The proteins enriched in extracellular vesicles participated in extracellular matrix remodeling, blood coagulation, inflammation, and angiogenesis [45]. Previous findings and our result indicated that ADSC-derived exosomal proteins might play a crucial role in tissue repair.

The KEGG pathway database was adopted to explore the enrichment of proteins, and the results revealed that several signaling pathways were related to proteins in ADSCs. Some of these pathways reflected the essential function of cells, such as metabolic pathways, regulation of actin cytoskeleton, ribosomes, and glycolysis. Thus, exosomes are derived from host cells and act as subcellular organelles. The results of KEGG analysis suggest that several enriched pathways, including the MAPK, VEGF, and Jak-STAT signaling pathways, are related to tissue repair. Previous studies have shown that adipose-derived stem cell exosomes alleviate neural injury via the MAPK pathway [20]. Moreover, the STAT3 pathway is involved in the ADSC exosome-induced autophagy and reduction of TGF-*β*1-induced hepatic fibrosis [25]. Activation of the VEGF/VEGF-R signaling pathway in exosomes can enhance angiogenesis, which is beneficial in tissue regeneration [24].

According to our knowledge, more than half of our cognition of exosome owes to the studies carried over the past few years. It became clear that the MSC-derived exosomes demonstrated great potentials for application in tissue repair, which is partially related to various exosome contents. Our results revealed that ADSC exosomes containing functional proteins were potential targets of new therapeutic methods for tissue repair. Our exosomal proteomic data might provide new clues and directions for future research and clinical application. Further detailed functional studied should be conducted to verify the functional roles of these candidate molecules and pathways because we have yet to fully understand the molecular composition of exosomes and mechanisms.

## 5. Conclusions

In summary, we demonstrated that a remarkable number of proteins involved in various biological processes are packed with ADSC-derived exosomes by employing various analytical techniques, including LC-MS/MS analyses. This work presented potential tissue repair-related proteins and signaling pathways and provided a rich proteomic data resource that might be valuable for future studies on tissue repair.

## Data Availability

The data used to support the findings of this study are available from the corresponding authors upon request.

## Supplementary Materials

Supplementary Materials The raw data files generated by LC-MS/MS during the current study are attached with the Excel file.
